# Clinical significance of preoperative nutrition and inflammation assessment tools in gastrointestinal cancer patients undergoing surgery: a retrospective cohort study

**DOI:** 10.3389/fnut.2025.1551048

**Published:** 2025-05-19

**Authors:** Valentina Casalone, Sara Erika Bellomo, Enrico Berrino, Simona Bo, Enrica Favaro, Alfredo Mellano, Elisabetta Fenocchio, Caterina Marchiò, Anna Sapino

**Affiliations:** ^1^Clinical Nutrition and Dietetics Unit, Candiolo Cancer Institute, FPO-IRCCS, Candiolo, Italy; ^2^Pathology Unit, Candiolo Cancer Institute, FPO-IRCCS, Candiolo, Italy; ^3^Department of Medical Sciences, University of Turin, Turin, Italy; ^4^Colorectal Surgical Unit, Candiolo Cancer Institute, FPO-IRCCS, Candiolo, Italy; ^5^Department of Medical Oncology, Candiolo Cancer Institute, FPO-IRCCS, Candiolo, Italy

**Keywords:** nutritional tools, malnutrition, inflammation, gastrointestinal cancer, cancer surgery, nutritional status, inflammatory status

## Abstract

**Background:**

Malnutrition and inflammation are associated with poorer surgical outcomes in patients with gastrointestinal cancer. However, it is still debated which parameters should be used to assess nutritional and inflammatory status. The aim of the present study was to investigate the prognostic role of specific parameters in predicting postoperative outcomes in this specific subgroup of patients.

**Methods:**

This retrospective study included 391 adult patients. Malnutrition risk, was assessed by preoperative validated Malnutrition Universal Screening Tool (MUST) score ≥2, lymphocyte count <900 n/mm^3^, albumin value <3.5 g/dL or a combination of the previous two parameters, the Prognostic Nutritional Index (PNI) < 45; inflammation was evaluated using preoperative Neutrophil-to-Lymphocyte Ratio (NLR) > 5, Platelet-to-Lymphocyte Ratio (PLR) > 150 and Lymphocyte-to-Monocyte Ratio (LMR) < 5. Statistical analysis was carried out using Univariate and Multivariate Analysis and General Linear Models.

**Results:**

Patients with higher preoperative MUST score (*p* < 0.0001), lower albumin level (*p* = 0.0002) or lower PNI (*p* = 0.002) had a greater need for parenteral nutrition support and a longer hospital stay was reported in patients with higher MUST score (*p* < 0.0001), lower albumin (*p* < 0.0001), lower PNI (*p* = 0.0002), higher NLR (*p* = 0.005) or lower LMR (*p* = 0.027). Complications were more common in patients with a higher MUST score (*p* = 0.029), lower albumin (*p* = 0.008) or lower PNI (*p* = 0.006). A MUST score ≥ 1 or a PNI < 45 was associated with a two-fold risk of postoperative complications (*p* = 0.008; *p* = 0.001), whereas albumin levels <35 g/L were correlated with a Three-fold risk of postsurgical complications (*p* = 0.008). OS was also worse in patients with higher MUST score (*p* = 0.004), PNI (*p* = 0.031) or NLR (*p* = 0.0002), with a three-fold risk of not surviving at 1 year in patients with a MUST score ≥2 (*p* = 0.003) or NLR ≥ 5 (*p* = 0.0003). Using general linear models for repeated measures, a preoperative MUST score >1 or albumin levels < 35 mg/dL was associated with lower postoperative erythrocyte cells and hemoglobin levels. Multivariate analysis confirmed MUST score, PNI and NLR as independent prognostic factors for survival or postoperative complications.

**Conclusion:**

The presence of preoperative malnutrition and/or inflammation is associated with worse postoperative outcomes in patients with gastrointestinal cancer. Early nutritional assessment, including all the above parameters, may allow more tailored intervention to reduce the risk of adverse postoperative outcomes.

## 1 Introduction

Surgery is the gold standard treatment for many non-advanced forms of gastrointestinal cancer (GC), and the number of operations is expected to increase to 45 million per year by 2030 (1). Malnutrition, is commonly reported in these patients on admission to hospital (2, 3), particularly in patients older than 70 years (4–6): weight loss occurring from malnutrition can be related to reduced nutrient intake or to the presence of a pathological process inducing muscle catabolism, known as cachexia, which is accompanied by inflammation (7). Many studies have reported that these conditions in patients undergoing GC surgery are associated with adverse postoperative outcomes (in terms of morbidity and mortality), altered immune responses, impaired wound healing and poor quality of life (8). Guidelines recommend the early identification of patients at risk and consecutive nutritional intervention; but currently there are no clear and unambiguous definitions of malnutrition and inflammation in the literature (6, 9–11). The European Society of Clinical Nutrition and Metabolism (ESPEN) and the Italian Society of Artificial Nutrition and Metabolism (SINPE) recommend the use of validated nutritional screening tools, such as the Nutritional Risk Screening 2002 (NRS 2002) or the Malnutrition Universal Screening Tool (MUST) (9, 11, 12). Other nutritional parameters that may reflect a state of malnutrition include serum albumin and lymphocyte count, either alone (13, 14) or as a combined score (the prognostic nutritional index, PNI), which is a marker of both nutritional and inflammatory status (15). Systemic inflammation, which is related to the development of many tumors (16) and can be modulated by the consumption of specific immunonutrients (17–20) can be measured by combining individual circulating markers into scores such as neutrophil-lymphocyte ratio (NLR), platelet-lymphocyte ratio (PLR), and lymphocyte-monocyte ratio (LMR) (21–23). Although recent studies have explored the use of some nutritional and inflammatory tools in different oncological settings, further studies are needed to implement their use in the daily clinical practice (24). A better understanding of the prognostic role of these parameters, which is the aim of the present study, could help to implement more precise and tailored nutritional interventions for cancer patients undergoing GC surgery.

## 2 Materials and methods

### 2.1 Objective

The aim of this observational study was to evaluate and compare the prognostic role of MUST score, lymphocyte count, albumin value, PNI, NLR, PLR and LMR in postsurgical outcomes of gastrointestinal cancer patients undergoing surgical treatment.

### 2.2 Study design and setting

This is a retrospective cohort study. Data were collected from the medical records of the Candiolo Cancer Institute FPO-IRCCS, in Italy. The study included adult patients consecutively admitted to the hospital between June 2019 and June 2021 for planned resective surgery for gastrointestinal cancers. Data deriving from the medical records of each patient were analyzed from the preoperative exams to the first year after surgery. The timeline of the study is shown in Supplementary Figure 1. This timeline is structured around 4 key timepoints for each patient: pre-surgery (median time 8 days before surgery), surgery, hospital discharge (median time 8 days post-surgery) and 1 year follow up for OS. At each timepoint, we collected several information, reported in the figure. At baseline and at hospital discharge, we collected several nutrition information, which are reported in Supplementary Figure 1 and in the Data Collection section. Considering inflammation parameters, we used the single pre-operative hematic withdraw for baseline characteristics, whereas after surgery we used the 3 longitudinal hematic withdraws routinely performed by clinicians. The study was approved by the Institute’s Ethics Committee and written informed consent was obtained from each patient for the use of their medical records (after hospital admissions) or, if the patient was unavailable, a substitute declaration was obtained regarding the retrospective nature of the study.

### 2.3 Characteristics of participants

Inclusion criteria (patients over 18 years of age with a diagnosis of gastro-esophageal, liver, pancreatic or colorectal cancer undergoing surgery) were based on previous studies (25–27). Patients undergoing emergency surgery were excluded for the higher risk of presenting postsurgical complications; laparoscopic surgery were excluded due to the exploratory nature of the procedure.

### 2.4 Data collection

Nutritional status was defined using the validated MUST screening, which has been shown to have higher accuracy (in terms of both sensitivity and specificity) in detecting malnutrition in hospitalized patients compared with other validated scores (28). MUST score is calculated by combining the body mass index (BMI), unintentional weight loss in the past 3–6 months and the potential acute effect of illness on food intake. The final score is 0 for well-nourished patients, 1 for patients at risk of malnutrition and 2 or more for malnourished patients (29). MUST screening was performed by dietitians or nurses before surgery. Other parameters used to assess nutritional status were preoperative level of albumin level (hypoalbuminemia <3.5 g/dL) (30, 31), and lymphocyte count [<900 n/mm^3^ (14)]. These two measures were also used to define the PNI, calculated as: serum albumin (g/L) + (5 × lymphocyte count ×10^9^/L). The PNI has been identified as a more accurate indicator of nutritional status and systemic immune competence than other variables and it has been shown to be an independent prognostic predictor in many malignant cancers (32). Patients with a PNI between 45 and 50 are considered at risk for malnutrition, whereas a PNI < 45 defines malnutrition (33). Systemic inflammation was assessed by NLR, PLR and LMR. Due to the heterogeneity of the literature, we selected similar studies to define cut off of these parameters for which we assigned the following values to determine the presence of inflammation: NLR > 5, PLR > 150 and LMR < 5 (21–23). Primary outcome variables were duration of oral fasting, need for and duration of nutritional support (enteral/parenteral), length of hospital stay (number of days from procedure to discharge), rate of postoperative complications, 1-year overall survival (OS). Postoperative complications were defined as any deviation from the normal course of recovery after surgery (i.e., anemia, low oxygen saturation, anastomotic leak, etc.). Exploratory outcomes were longitudinal changes in white blood cells, red blood cells, hemoglobin, platelets, and total protein (from baseline to first, third and fifth postoperative day – POD).

### 2.5 Statistical analysis

Continuous data are reported as mean ± standard deviation and categorical variables are presented as frequencies and percentages. In detail, data on hematologic parameters and their dynamics, duration of fasting/nutritional support, duration of hospital stay are reported as median ± standard deviation. Data on prevalence of malnutrition, use of nutritional support, complications and overall survival are classified on a dichotomous scale (yes/no) and reported as absolute frequencies and percentages in brackets. Statistical analyses were performed dividing patients into groups according to literature-based cutoff values of nutritional and inflammatory assessment tools. Differences in categorical data were assessed using the χ^2^ test. For continuous variables, normality was tested using the D’Agostino test. As most parameters did not follow a Gaussian distribution, differences between two groups were assessed using the Mann–Whitney U test and, for more than two groups, the Kruskal-Wallis test. Backward stepwise logistic regression was performed with the dichotomous classification of: MUST (score ≥2), lymphocyte count, albumin, PNI (>50), NLR, PLR and LMR values. Independent variables were selected as baseline or postsurgical clinical assessments that were statistically significant in the corresponding univariable setting. Variables found to be statistically significant in the univariate analysis were further examined in a multivariate analysis. General linear models (GLM) repeated measures test was performed to analyze the dynamics of hematologic parameters over time in the different patient groups stratified according to MUST and albumin cut-off values. The relative influence of some parameters was expressed as odd ratios (ORs) for having postoperative complications or dying in the first year after surgery. Statistical significance was determined using an alpha level of 0.05 and two-sided tests. All statistical analyses were performed using the SPSS statistical software program, version 25.0.

## 3 Results

### 3.1 Cohort description

We collected a total of 391 patients (232 men, and 159 women) with GC, with a mean age of 65 years. More than half of the patients (224/391, 57%) had colorectal cancer (CRC), 23% had gastrointestinal metastatic cancer (M+, 91/391), 13% had gastroesophageal cancers (UGI, 51/391), 4% hepatobiliary cancer (HPB, 16/391) and 2% pancreatic cancers (PAN, 9/391). The stage of the cancer was as follows: stage I 33% (126/391), stage II 20% (75/391), stage III 23% (87/391), and stage IV 23% (90/391), while the remaining 2% (7/391) cancer could not be classified into any stage after histologic examination. The ASA Physical Status Classification System score was 1 in 3% of patients (11/391), 2 in 53% (206/391), 3 in 39% (154/391) and 4 in 5% (20/391). Surgery was the first treatment option for 65% of patients (253/391), while 35% had received neoadjuvant treatment (138/391). Mini-invasive surgery was performed in 43% of patients (171/391). According to the BMI, 3% of patients were classified as underweight (10/391), 49% as normal weight (194/391), 31% as overweight (120/391) and 17% as obese (67/391). The preoperative MUST score was not assessed in 7% (29/391) of patients, while 71.4% (279/391) had a score of 0 (well nourished), 12% (47/391) had a score of 1 (at risk of malnutrition) and 9% had a score of 2 or more (36/391 malnourished). Only 3% (10/391) of patients received preoperative nutritional support (oral in 50%, parenteral in 30%, and combined oral-parenteral in 20%). Similarly, only 2% (8/391) of patients received immune-enriched nutrition in the perioperative period, whereas 25% (96/391) received maltodextrins for 2 days prior to surgery. The mean postoperative fasting period was 1.8 days: 24% (93/391) and 2% (8/391) of patients required parenteral (PN) and enteral nutrition (EN), respectively. The mean hospital stay was 6.6 days; 28% (111/391) of patients had postoperative complications, including 39% (43/111) mechanical, 22% (24/111) hematologic, 4% (5/111) cardiac, 13% (14/111) pulmonary, 13% (15/111) multiple, and 9% (10/111) other complications.

### 3.2 Pre-operative baseline nutrition assessment tools

**Figure 1:**
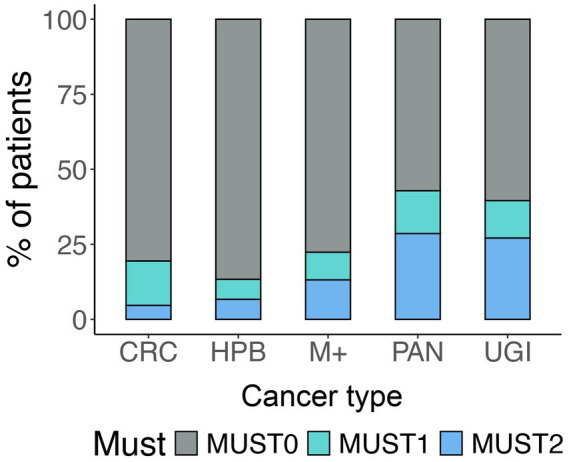
MUST score and cancer sites. The figure represents the prevalence of different MUST scores for each cancer site.

Baseline characteristics of patients according to the nutritional tools are described in Table 1. First, we reported a total of 36/362 (10%) malnourished patients according to the MUST score cut-off, in line with lymphocyte count (41/378, 11%) and with albumin level (28/367, 8%). By combing the latter parameters into the PNI value, we reported a higher malnutrition rate (80/367, 22%). Males (*p* = 0.03) and older patients (*p* < 0.001) had higher levels of malnutrition when considering PNI, but sex polarization was not confirmed by the MUST score (*p* < 0.01). Albumin and PNI correlated with ASA score (*p* = 0.046 and *p* < 0.001, respectively). The MUST also stratified cancer type by level of malnutrition: UGI (27% by MUST score and 18% by albumin levels) and PAN (29% by MUST score and 22% by albumin) were significantly malnourished (*p* < 0.001, Figure 1). Finally, we found no association between malnutrition and cancer stage. Finally, the neoadjuvant treatment did not seem to influence MUST score, albumin and PNI (Table 1).

**Table 1:** Baseline characteristics of patients according to the nutritional assessment tools.

Parameters		MUST score [NA = 29]				Lymphocytes [NA = 13]			Albumin [NA = 24]			PNI [NA = 24]			
		0 (279)	1 (47)	≥2 (36)	*p*-value	>0.9 (337)	<0.9 (41)	*p*-value	>35 (339)	<35 (28)	*p*-value	>50 (161)	45–50 (126)	<45 (80)	*p*-value
Prevalence	%	77	13	10	–	90	10	–	92.4	7.6	–	43.9	34.3	21.8	–
Sex	Male	177 (83)	23 (11)	13 (6)	**0.002**	199 (89)	25 (11)	0.813	203 (93)	16 (7)	0.776	90 (41)	71 (32)	58 (27)	**0.030**
	Female	102 (69)	24 (16)	23 (15)		138 (89)	16 (11)		136 (92)	12 (8)		71 (48)	55 (37)	22 (15)	
Comorbidities	Yes	213 (76)	37 (14)	29 (10)	0.817	256 (88)	34 (12)	0.319	257 (91)	25 (9)	0.104	121 (43)	95 (34)	66 (23)	0.398
	No	66 (80)	10 (12)	7 (8)		81 (92)	7 (8)		82 (97)	3 (3)		40 (47)	31 (37)	14 (16)	
Age	Years	66 ± 12.1	66.1 ± 13.6	64.3 ± 12.2	0.671	65.3 ± 12.4	66 ± 11.5	0.850	65.1 ± 12.1	69.5 ± 13.6	**0.048**	62.1 ± 12	66.5 ± 11.9	70.4 ± 11.4	**<0.00001**
Cancer type	UGI	29 (60)	6 (13)	13 (27)	**0.0004**	43 (86)	7 (14)	0.209	41 (82)	9 (18)	**0.010**	24 (48)	13 (26)	13 (26)	0.627
	HBP	13 (87)	1 (7)	1 (6)		15 (94)	1 (6)		15 (100)	0 (0)		8 (54)	5 (33)	2 (13)	
	PAN	4 (57)	1 (14)	2 (29)		9 (100)	0 (0)		7 (78)	2 (22)		3 (33)	3 (33)	3 (34)	
	CRC	174 (81)	32 (15)	10 (4)		184 (87)	28 (13)		192 (94)	13 (6)		82 (40)	76 (37)	47 (23)	
	M+	59 (78)	7 (9)	10 (13)		86 (94)	5 (6)		84 (95)	4 (5)		44 (50)	29 (33)	15 (17)	
Cancer stage	0	3 (60)	1 (20)	1 (20)	0.214	5 (83)	1 (17)	0.288	6 (100)	0 (0)	0.279	4 (66)	1 (17)	1 (17)	0.124
	I	95 (81)	12 (10)	11 (9)		110 (89)	13 (11)		109 (92)	10 (8)		57 (48)	41 (34)	21 (18)	
	II	60 (82)	8 (11)	5 (7)		59 (87)	9 (13)		61 (92)	5 (8)		23 (35)	25 (38)	18 (27)	
	III	55 (66)	18 (21)	11 (13)		71 (85)	13 (15)		72 (88)	10 (12)		29 (35)	27 (33)	26 (32)	
	IV	60 (80)	7 (9)	8 (11)		85 (94)	5 (6)		84 (97)	3 (3)		43 (49)	31 (36)	13 (15)	
	NA	[6]	[1]	[0]		[7]	[0]		[7]	[0]		[156]	[125]	[79]	
ASA score	1	9 (82)	2 (18)	0 (0)	0.378	10 (100)	0 (0)	0.605	9 (100)	0 (0)	**0.046**	6 (67)	3 (33)	0 (0)	**0.00001**
	2	144 (78)	20 (11)	21 (11)		182 (90)	21 (10)		189 (95)	9 (5)		96 (48)	74 (37)	28 (15)	
	3	114 (78)	20 (14)	12 (8)		129 (88)	17 (12)		125 (89)	16 (11)		56 (41)	44 (31)	41 (28)	
	4	12 (60)	5 (25)	3 (15)		16 (84)	3 (16)		16 (84)	3 (16)		3 (16)	5 (26)	11 (58)	
MUST score	0	–	–	–	–	243 (91)	25 (9)	0.233	242 (93)	18 (7)	0.125	112 (43)	93 (36)	55 (21)	0.723
	1	–	–	–		37 (82)	8 (18)		40 (93)	3 (7)		19 (44)	13 (30)	11 (26)	
	≥ 2	–	–	–		32 (89)	4 (11)		30 (83)	6 (17)		14 (40)	11 (30)	11 (30)	
	NA	–	–	–		[25]	[4]		[27]	[1]		[16]	[9]	[3]	
BMI		26.6 ± 4.3	23.3 ± 4.4	23.9 ± 5.9	**<0.00001**	25.9 ± 4.6	26.1 ± 5	0.944	26 ± 4.7	25.5 ± 3.9	0.891	26 ± 4.8	25.6 ± 4.3	26.4 ± 5	0.587
Preoperative nutritional support	Yes	4 (40)	1 (10)	5 (50)	**0.0001**	9 (90)	1 (10)	0.930	8 (80)	2 (20)	0.135	2 (20)	3 (30)	5 (50)	0.076
	No	275 (78)	46 (13)	31 (9)		328 (89)	40 (11)		331 (93)	26 (7)		159 (45)	123 (34)	75 (21)	
Baseline biochemical exams	WBC	6.6 ± 2.1	7.9 ± 3.5	7.7 ± 3.7	0.057	7 ± 2.4	6 ± 3.4	**0.00038**	6.6 ± 2.3	8.8 ± 2.8	**<0.00001**	6.9 ± 2	6.2 ± 2.3	7.3 ± 3.1	**0.002**
	Lymphocytes	1.6 ± 0.7	1.6 ± 0.6	1.6 ± 0.7	0.685	1.7 ± 0.7	0.7 ± 0.1	**<0.00001**	1.7 ± 0.7	1.3 ± 0.8	**0.00528**	2.1 ± 0.8	1.4 ± 0.4	1.1 ± 0.4	**<0.00001**
	Hemoglobin	12.7 ± 1.8	12.3 ± 1.9	12.3 ± 1.9	0.270	12.8 ± 1.8	12.1 ± 1.7	**0.016**	12.9 ± 1.7	11 ± 2	**<0.00001**	13.4 ± 1.7	12.6 ± 1.6	11.6 ± 2	**<0.00001**
	Albumin	40.8 ± 3.5	40 ± 4	39.6 ± 4.5	0.120	40.9 ± 3.6	38.5 ± 4	**0.0004**	41.4 ± 2.8	32.1 ± 2.4	**<0.00001**	43 ± 2.4	40.7 ± 2	36 ± 3.3	**<0.00001**
	PNI	48.9 ± 5.4	48.2 ± 6	47.7 ± 6.1	0.648	49.7 ± 5.3	42 ± 4.1	**<0.00001**	49.7 ± 4.9	38.8 ± 4.2	**<0.00001**	53.6 ± 3.8	47.6 ± 1.5	41.3 ± 3.2	**<0.00001**
	NLR	3.1 ± 2.5	4.6 ± 6	3.5 ± 2.1	0.152	2.8 ± 1.6	7.6 ± 7	**<0.00001**	3 ± 2.7	6.3 ± 4.5	**<0.00001**	2 ± 0.9	3 ± 1.2	6 ± 5.2	**<0.00001**
	PLR	170.5 ± 90.1	202.5 ± 137.2	185.7 ± 92.5	0.263	154.6 ± 73	73 ± 128.1	**<0.00001**	167.4 ± 92.8	237.9 ± 113.1	**0.0001**	121.1 ± 50	182.9 ± 78.7	261 ± 120.4	**<0.00001**
	LMR	3 ± 1.4	3 ± 1.4	2.7 ± 1.3	0.517	3.1 ± 1.4	1.7 ± 0.6	**<0.00001**	3 ± 1.4	2.1 ± 1.1	**0.0001**	3.8 ± 1.5	2.6 ± 1	1.9 ± 0.8	**<0.00001**
Neoadjuvant CT/RT	Yes	89 (75)	13 (11)	16 (14)	0.237	114 (83)	23 (17)	**0.005**	124 (93)	10 (7)	0.927	54 (40)	50 (38)	30 (22)	0.551
	No	190 (78)	34 (14)	20 (8)		223 (92)	18 (8)		215 (92)	18 (8)		107 (46)	76 (33)	50 (21)	
Mini-invasive surgery	Yes	132 (82)	18 (11)	11 (7)	0.108	152 (92)	14 (8)	0.182	155 (97)	6 (3)	**0.013**	70 (43)	60 (37)	31 (20)	0.454
	No	147 (73)	29 (15)	25 (12)		185 (87)	27 (13)		184 (89)	22 (11)		91 (44)	66 (32)	49 (24)	

Statistically significant results are highlighted in bold.

### 3.3 Post-operative outcomes according to the nutrition assessment tools

**Figure 2:**
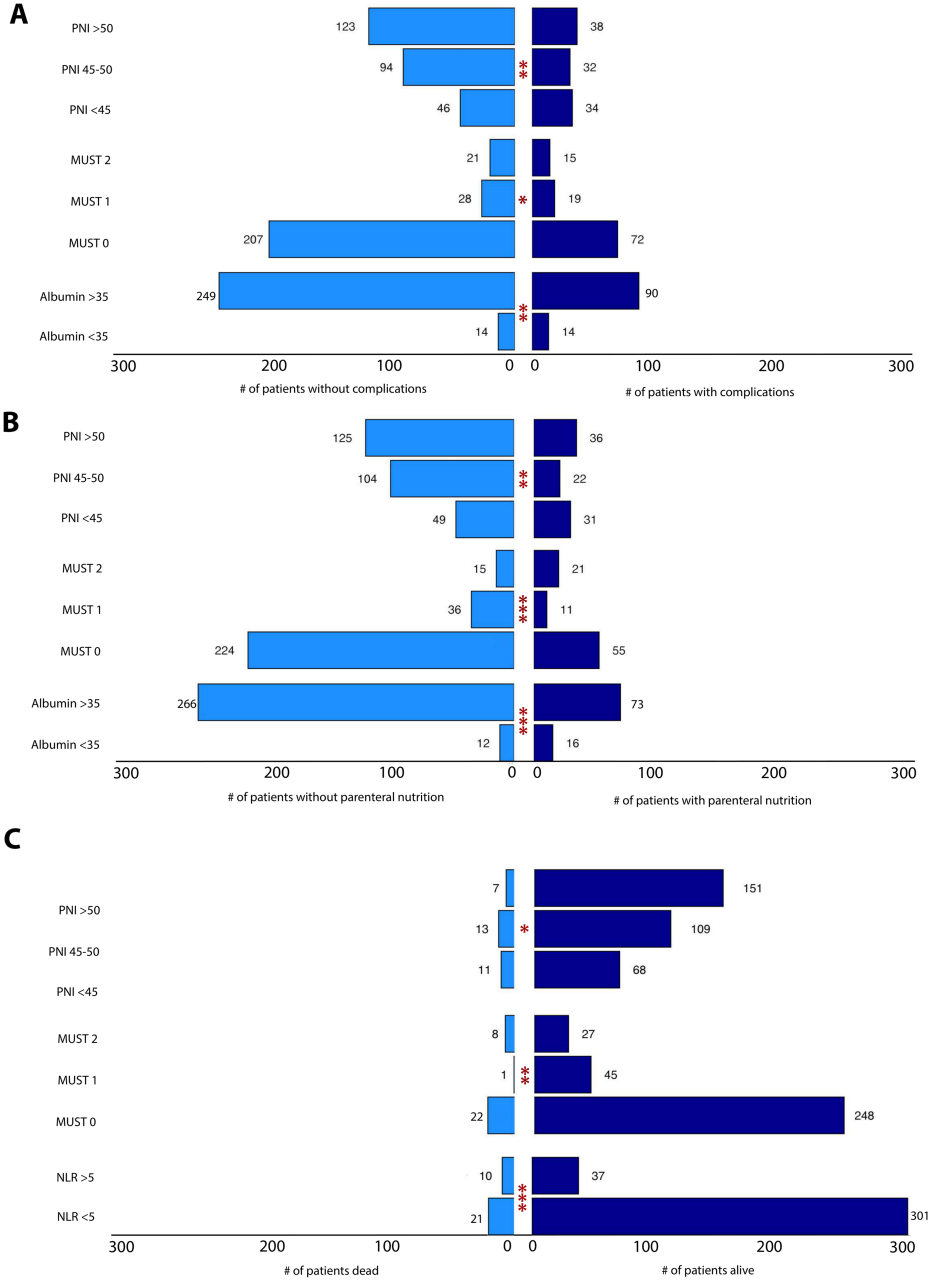
**(A)** Association between nutrition assessment tools and complications. The tornado plot represents the statistically significant associations between some nutritional parameters (PNI, MUST score, albumin) and postoperative complications. **(B)** Association between nutrition assessment tools and PN need. The tornado plot represents the statistically significant associations between some nutritional parameters (PNI, MUST score, albumin) and PN need. **(C)** Association between nutrition and inflammation assessment tools and 1-year OS. The tornado plot represents the statistically significant associations between some parameters (PNI, MUST score, NLR) and 1 year-OS. *p < 0.05, **p < 0.01, ***p < 0.001.

Postoperative outcomes of patients according to the nutritional tools are shown in Table 2. The MUST score was significantly associated with all factors examined. Specifically, patients with preoperative malnutrition had a longer hospital stay (9.5 days vs. 6.5 days), more postoperative complications (Figure 2A) even with a score ≥ 1 (OR = 1.995, *p* = 0.008) or death during the first year of FU (OR = 3.593 *p* = 0.003 with a score ≥2; Figure 2C). Similarly, also a lower PNI score correlated with the worst postoperative outcomes (Figure 2 and Table 2), except for the EN parameters: a score <45 was associated with a 2-fold higher risk of postoperative complications (OR = 2.291, *p* = 0.001). Albumin overlapped significantly with the PNI results [with an OR = 2.797 (*p* = 0.008) of postoperative complications for baseline levels <35 g/L], whereas no correlation was found between lymphocyte count and postoperative events (Table 2).

**Table 2:** Post-surgical outcomes according to the nutritional assessment tools.

Parameters		MUST score [NA = 29]				Lymphocytes [NA = 13]			Albumin [NA = 24]			PNI [NA = 24]			
		0 (279)	1 (47)	≥2 (36)	*p*-value	>0.9 (337)	<0.9 (41)	*p*-value	>35 (339)	<35 (28)	*p*-value	>50 (161)	45–50 (126)	<45 (80)	*p*-value
Fasting length	Days	1.6 ± 1.7	2 ± 2.4	3.6 ± 4.8	**0.000**	1.8 ± 2.3	2.1 ± 2.1	0.113	1.7 ± 2.3	3.4 ± 2.7	**0.000**	1.9 ± 2.8	1.5 ± 1.7	2.2 ± 2.2	**0.028**
PN need	Yes	55 (63)	11 (13)	21 (24)	**<0.00001**	80 (87)	12 (13)	0.436	73 (82)	16 (18)	**0.00002**	36 (40)	22 (25)	31 (35)	**0.002**
	No	224 (81)	36 (13)	15 (6)		257 (90)	29 (10)		266 (96)	12 (4)		125 (45)	104 (37)	49 (18)	
PN length	Days	1.4 ± 3.7	1.6 ± 4	4.4 ± 6.5	**0.000**	1.6 ± 3.9	2.4 ± 5.3	0.286	1.4 ± 3.6	5.5 ± 6.9	**0.000**	1.6 ± 4.3	1 ± 2.8	2.9 ± 5.1	**0.000**
EN need	Yes	2 (24)	3 (38)	3 (38)	**0.002**	8 (100)	0 (0)	0.319	7 (88)	1 (12)	0.600	4 (50)	2 (25)	2 (25)	0.854
	No	277 (78)	44 (13)	33 (9)		329 (89)	41 (11)		332 (92)	27 (8)		157 (44)	124 (34)	78 (22)	
EN length	Days	0.1 ± 0.6	0.4 ± 1.7	0.7 ± 3	**0.006**	0.2 ± 1.3	0 ± 0.2	1	0.2 ± 1.3	0.1 ± 0.6	0.706	0.2 ± 1.5	0.2 ± 1.3	0.1 ± 0.6	0.640
Hospital Stay	Days	6.3 ± 4.7	6.8 ± 5.6	9.5 ± 7.1	**0.000**	6.5 ± 5	7.6 ± 5.6	0.103	6.4 ± 4.9	9.9 ± 6.2	**<0.0001**	6.3 ± 4.7	6 ± 4.5	8.4 ± 6.4	**0.0002**
Complications	Yes	72 (68)	19 (18)	15 (14)	**0.029**	94 (88)	13 (12)	0.609	90 (87)	14 (13)	**0.008**	38 (37)	32 (31)	34 (32)	**0.006**
	No	207 (81)	28 (11)	21 (8)		243 (90)	28 (10)		249 (95)	14 (5)		123 (47)	94 (36)	46 (17)	
1-year OS	Yes	248 (78)	45 (14)	27 (8)	**0.004**	302 (89)	36 (11)	0.866	306 (93)	22 (7)	0.057	151 (46)	109 (33)	68 (21)	**0.031**
	No	22 (71)	1 (3)	8 (26)		28 (90)	3 (10)		26 (84)	5 (16)		7 (23)	13 (42)	11 (35)	
	[Tot]	[270]	[46]	[35]		[330]	[39]		[332]	[27]		[158]	[122]	[79]	

Statistically significant results are highlighted in bold.

### 3.4 Pre-operative baseline inflammation assessment tools

Baseline characteristics of patients according to the inflammatory parameters are shown in Table 3. Each of the inflammatory proxies (NLR > 5, PLR > 150 and LMR < 5) stratified the cohort into independent groups enriched for specific features (*p* < 0.0001). Lower LMR was significantly detected in older (*p* = 0.008) and male patients (*p* = 0.003); whereas higher PLR was significantly associated with cancer type (*p* = 0.0001) and stage (*p* = 0.001) and NLR was significantly correlated with worst ASA score (*p* = 0.007) and MUST (*p* = 0.032). Notably, all three inflammatory parameters were statistically associated with all the baseline biochemical parameters analyzed, whereas neoadjuvant treatment was not correlated with any inflammatory status.

**Table 3:** Baseline characteristics of patients according to the inflammation assessment tools.

Parameters		NLR [NA = 13]			PLR [NA = 13]			LMR [NA = 13]		
		<5 (330)	≥5 (48)	*p*-value	<150 (191)	≥150 (187)	*p*-value	≥5 (30)	<5 (348)	*p*-value
Prevalence	%	87	13	–	50.5	49.5	–	7.9	92.1	**–**
Sex	Male	193 (86)	31 (14)	0.422	117 (52)	107 (48)	0.424	10 (4)	214 (96)	**0.003**
	Female	137 (89)	17 (11)		74 (48)	80 (52)		20 (13)	134 (87)	
Comorbidities	Yes	249 (85)	41 (15)	0.127	148 (51)	142 (49)	0.721	22 (8)	268 (92)	0.647
	No	81 (92)	7 (8)		43 (49)	45 (51)		8 (9)	80 (91)	
Age	Years	65 ± 12.2	68.4 ± 12.4	0.092	64.9 ± 12.3	65.9 ± 12.3	0.521	60.3 ± 10.2	65.9 ± 12.4	**0.008**
Cancer type	UGI	42 (84)	8 (16)	0.176	27 (54)	23 (46)	**0.0001**	5 (10)	45 (90)	0.427
	HBP	16 (100)	0 (0)		13 (81)	3 (19)		3 (19)	13 (81)	
	PAN	7 (78)	2 (22)		5 (56)	4 (44)		0 (0)	9 (100)	
	CRC	181 (85)	31 (15)		86 (41)	126 (59)		15 (7)	197 (93)	
	M+	84 (92)	7 (8)		60 (66)	31 (34)		7 (8)	84 (92)	
Cancer stage	0	6 (100)	0 (0)	0.051	4 (67)	2 (33)	**0.001**	1 (17)	5 (83)	0.088
	I	111 (90)	12 (10)		64 (52)	59 (48)		15 (12)	108 (88)	
	II	56 (82)	12 (18)		27 (40)	41 (60)		4 (6)	64 (94)	
	III	67 (80)	17 (20)		32 (84)	52 (16)		2 (2)	82 (98)	
	IV	83 (92)	7 (8)		60 (67)	30 (33)		6 (7)	84 (93)	
	NA	[7]	[0]		[4]	[3]		[2]	[5]	
ASA score	1	10 (100)	0 (0)	**0.007**	7 (70)	3 (30)	0.577	1 (10)	9 (90)	0.476
	2	185 (91)	18 (9)		99 (46)	104 (54)		19 (9)	184 (91)	
	3	122 (83)	24 (17)		76 (52)	70 (48)		10 (7)	136 (93)	
	4	13 (68)	6 (32)		9 (47)	10 (53)		0 (0)	19 (100)	
MUST	0	241 (90)	27 (10)	**0.032**	138 (51)	130 (49)	0.488	23 (9)	245 (91)	0.328
	1	35 (78)	10 (22)		21 (47)	24 (53)		2 (4)	43 (96)	
	≥2	29 (81)	7 (19)		15 (42)	21 (58)		1 (3)	35 (99)	
	NA	[25]	[4]		[17]	[12]		[4]	[25]	
BMI		26 ± 4.6	25.4 ± 4.8	0.192	26.1 ± 4.9	25.7 ± 4.4	0.632	25.8 ± 4.3	25.9 ± 4.7	0.727
Preoperative nutritional support	Yes	7 (70)	3 (30)	0.096	5 (50)	5 (50)	0.973	1 (10)	9 (90)	0.807
	No	322 (88)	45 (22)		186 (51)	182 (49)		29 (79)	339 (21)	
Baseline biochemical exams	WBC	6.4 ± 1.9	9.9 ± 3.7	**<0.00001**	6.7 ± 2.2	7 ± 2.8	0.636	6.8 ± 2.9	6.9 ± 2.5	0.277
	Lymphocytes	1.7 ± 0.7	1.1 ± 0.5	**<0.00001**	2 ± 0.8	1.2 ± 0.4	**<0.00001**	2.8 ± 1.3	1.5 ± 0.6	**<0.00001**
	Hemoglobin	12.9 ± 1.8	11.1 ± 1.7	**<0.00001**	13.3 ± 1.6	12 ± 1.9	**<0.00001**	14.1 ± 1.5	12.6 ± 1.8	**<0.00001**
	Albumin	41.2 ± 3.3	36.7 ± 4.1	**<0.00001**	41.6 ± 3.2	39.7 ± 3.9	**<0.00001**	42 ± 3.1	40.6 ± 3.7	**0.016**
	PNI	49.8 ± 5.1	42 ± 5.1	**<0.00001**	51.6 ± 5.3	46 ± 4.6	**<0.00001**	55.9 ± 6.7	48.3 ± 5.2	**<0.00001**
	NLR	2.5 ± 1	8.7 ± 5.8	**<0.00001**	2.1 ± 0.9	4.6 ± 3.9	**<0.00001**	1.3 ± 0.4	3.5 ± 3.2	**<0.00001**
	PLR	153 ± 72.5	313.3 ± 126.1	**<0.00001**	106.1 ± 26.6	242.9 ± 95.1	**<0.00001**	86.4 ± 32.1	181.3 ± 97.6	**<0.00001**
	LMR	3.2 ± 1.4	1.6 ± 0.7	**<0.00001**	3.7 ± 1.6	2.2 ± 0.9	**<0.00001**	6.4 ± 1.6	2.7 ± 1	**<0.00001**
Neoadjuvant CT/RT	Yes	123 (90)	14 (10)	0.275	69 (50)	68 (50)	0.962	8 (6)	129 (94)	0.255
	No	207 (86)	34 (14)		122 (51)	119 (49)		22 (9)	219 (91)	
Mini-invasive surgery	Yes	151 (91)	15 (9)	0.058	80 (48)	86 (52)	0.421	15 (9)	151 (91)	0.484
	No	179 (84)	33 (16)		111 (52)	101 (48)		15 (7)	197 (93)	

Statistically significant results are highlighted in bold.

### 3.5 Post-operative outcomes according to the inflammation assessment tools

Postoperative outcomes of patients stratified by inflammatory parameters are described in Table 4. NLR was more predictive compared to PLR and LMR, showing a statistically significant correlation with both length of stay (*p* = 0.005) and 1-year overall survival (OS; *p* = 0.001; OR = 4.027, *p* = 0.0003 with a NLR ≥ 5). Only the PLR was associated with EN support (*p* = 0.035). Length of hospital stay was also statistically associated with LMR also (*p* = 0.027). Figure 2 summarizes the statistically significant associations between nutritional/inflammatory scores (PNI, MUST, Albumin and NLR) and three different outcomes: postoperative complications (Figure 2A), PN need (Figure 2B) and 1-year OS (Figure 2C).

**Table 4:** Post-surgical outcomes according to the inflammation assessment tools.

Parameters		NLR [NA = 13]			PLR [NA = 13]			LMR [NA = 13]		
		<5 (330)	≥5 (48)	*p*-value	<150 (191)	≥150 (187)	*p*-value	≥5 (30)	<5 (348)	*p*-value
Fasting length	Days	1.8 ± 2.4	1.9 ± 2	0.502	1.9 ± 2.6	1.8 ± 2	0.347	1.5 ± 1.5	1.9 ± 2.4	0.535
PN need	Yes	77 (84)	15 (16)	0.232	48 (52)	44 (48)	0.717	7 (8)	85 (92)	0.894
	No	253 (88)	33 (12)		143 (50)	143 (50)		23 (8)	263 (92)	
PN length	Days	1.7 ± 4.2	1.8 ± 3.2	0.186	2 ± 4.9	1.4 ± 3.1	0.704	4 (3)	2 (2)	0.853
EN need	Yes	8 (100)	0 (0)	0.276	7 (88)	1 (12)	**0.035**	0 (0)	8 (100)	0.401
	No	322 (87)	48 (13)		184 (50)	186 (50)		30 (8)	340 (92)	
EN length	Days	0.2 ± 1.3	0 ± 0.1	0.866	0.3 ± 1.7	0 ± 0.2	0.092	0 ± 0	0.2 ± 1.3	0.381
Hospital stay	Days	6.5 ± 5.1	7.8 ± 5	**0.005**	6.8 ± 5.6	6.5 ± 4.6	0.845	5.1 ± 3.3	6.8 ± 5.2	**0.027**
Complications	Yes	89 (83)	18 (17)	0.130	57 (53)	50 (47)	0.503	6 (6)	101 (94)	0.293
	No	241 (89)	30 (11)		134 (49)	137 (51)		24 (9)	247 (91)	
1-year OS	Yes	301 (89)	37 (11)	**0.001**	173 (51)	165 (19)	0.963	29 (9)	306 (91)	0.297
	No	21 (68)	10 (34)		16 (52)	15 (48)		1 (3)	32 (97)	
	[Tot]	(322)	(47)		[189]	[180]		[30]	[338]	

Statistically significant results are highlighted in bold.

### 3.6 Multivariate analysis and exploratory outcomes

**Figure 3:**
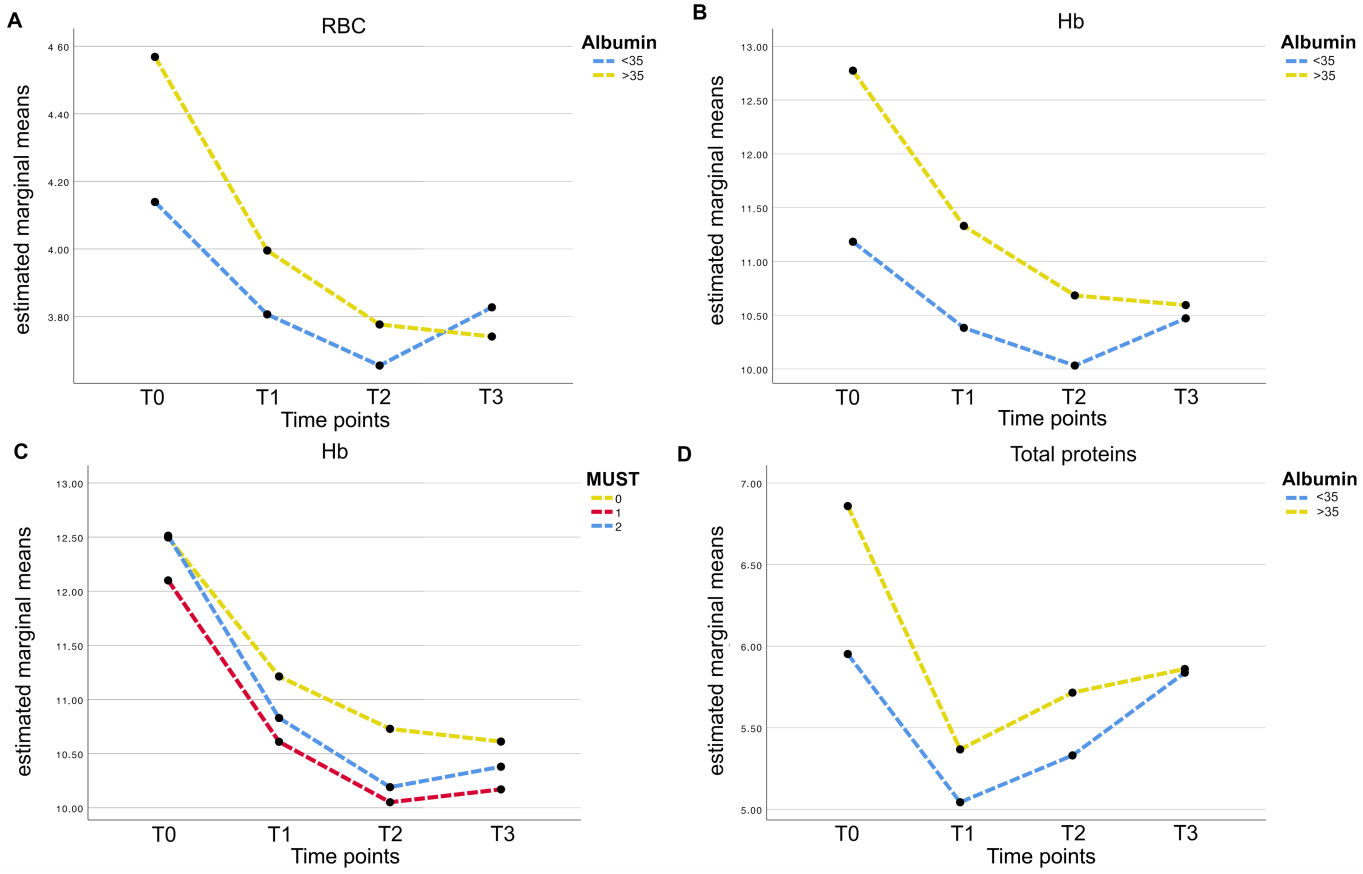
**(A)** RBC post-operative longitudinal variations (according to preoperative albumin levels). Medium values of RBC are presented at four time points (T0 = preoperative; T1 = POD1; T2 = POD3; T3 = POD5). Value of patients presenting a preoperative albumin value > 35 g/L are represented by the yellow line; whereas the blue line is for patients with preoperative albumin level < 35 g/L. **(B)** Hb post-operative longitudinal variations (according to preoperative albumin levels). Medium values of Hb are presented at four time points (T0 = preoperative; T1 = POD1; T2 = POD3; T3 = POD5). Value of patients presenting a preoperative albumin value > 35 g/L are represented by the yellow line; whereas the blue line is for patients with preoperative albumin level <35 g/L. **(C)** Hb post-operative longitudinal variations (according to preoperative MUST score). Medium values of Hb are presented at four time points (T0 = preoperative; T1 = POD1; T2 = POD3; T3 = POD5). Value of patients presenting a preoperative MUST score = 0 are represented by the yellow line; whereas blue line and red line are for patients with a preoperative MUST score of 1 and ≥ 2, respectively. **(D)** Total proteins post-operative longitudinal variations (according to preoperative albumin levels). Medium values of Total Protein are presented at four time points (T0 = preoperative; T1 = POD1; T2 = POD3; T3 = POD5). Value of patients presenting a preoperative albumin value > 35 g/L are represented by the yellow line; whereas the blue line is for patients with preoperative albumin level < 35 g/L.

Based on statistically significant associations in the univariable setting, multivariate analyses were performed considering different outcomes. The most clinically relevant outcomes, postoperative complications or 1-year overall survival are shown in Table 5. MUST (*p* = 0.008) and PNI (*p* < 0.001) were independent predictive factors of complications, whereas MUST score (*p* = 0.038), NLR (*p* = 0.001) and PLR (0.004) were independent prognostic factors for OS. Significant multivariate analyses with other nutritional/inflammatory parameters are shown in the Supplementary Table 1. We next analyzed the impact of longitudinal changes in biochemical parameters on nutritional/inflammatory outcomes. Notably, as shown in Figure 3, statistically significant longitudinal changes in biochemical parameters were demonstrated only when preoperative MUST score and albumin levels were used to split up the samples. Specifically, RBC, hemoglobin and total protein decreased in the first preoperative days, especially in patients reporting lower preoperative albumin levels (Figures 3A,B,D), whereas hemoglobin levels decreased more in patients reporting a preoperative MUST score of 1 or 2 (Figure 3C).

**Table 5:** Multivariate analysis of baseline nutritional and inflammatory parameters.

Parameters	Complications			1-year OS		
	Exp (B)	S.E.	*p*-value	Exp (B)	S.E.	*p*-value
MUST	1.460	0.142	**0.008**	0.662	0.199	**0.038**
Lymph	1.472	0.251	0.124	0.129	1.135	0.072
Alb	9.550	8.681	0.795	1.311	0.729	0.710
PNI	0.978	0.003	**0.000**	1.052	0.029	0.082
NLR	1.034	0.039	0.395	0.733	0.095	**0.001**
PLR	1.000	0.002	0.993	1.012	0.004	**0.004**
LMR	0.896	0.112	0.324	1.537	0.241	0.075

Statistically significant results are highlighted in bold.

## 4 Discussion

Recently, nutritional, and inflammatory status has been associated with oncologic outcomes in different settings. However, standardization of the biomarkers used to assess malnutrition is still lacking (9, 34–38). Our aim was to investigate the predictive role of the nutrition-based biomarkers in the scenario of gastrointestinal cancers.

In our study, the preoperative MUST score showed that 13% of patients were considered at risk of malnutrition, whereas 10% were already severely malnourished, which is similar to the prevalence reported by Almasaudi and colleagues (9). Higher rates of malnutrition have been reported in literature: this discrepancy could potentially be related to the application of different screening tools (for example the 2002 NRS score which analyses multiple factors, including the presence of disease) and to the enrolment of emergency surgery’s cases which usually report critical conditions (39). In addition, our cohort was mainly composed of patients with colorectal cancer, which is typically associated with lower rates of malnutrition (2). On the other hand, we reported a high MUST score in more than the previously described 40% of upper gastrointestinal and pancreatic cancer patients, with an impaired nutritional status (40).

Our results showed that a preoperative MUST score ≥2 was associated with worse clinical outcomes. A mean hospital stay >7 days and a worse OS were also reported by the Almasaudi study (9). Although preoperative nutritional support has been shown to be more common in malnourished patients, most of them (86%) did not receive it, probably due to lack of early nutritional screening (41). Furthermore, it is not surprising that a small percentage of patients with a MUST score of 0–1 received nutritional support: an early prescription of these products aims to reduce nutritional status impairment and these data could be the proof of their efficacy (2, 42). We also found that malnourished patients were associated with higher complication rates (MUST score ≥1) and lower survival (MUST score ≥2) calculated in the first year after surgery.

Alternative parameters such as lymphocyte count and albumin level can be used to assess nutritional status (13, 14). In our study lymphocyte count did not correlate with any postoperative outcomes, in contrast to the study by Yamamoto and colleagues that predicted OS in colorectal cancer patients by combining both pre- and postoperative lymphocyte count (43). On the other hand we have confirmed the role of serum albumin to define nutritional status (13, 44), by associating lower albumin level with age (45) longer hospital stay, complication rates and overall survival (46, 47). We also found that malnourished patients, identified by the albumin level, required greater use of nutritional support in the postoperative period. Finally, a high PNI score, which combines both nutritional and inflammatory status, identified patients with a greater number of postoperative complications and reduced overall survival, in agreement with previous reports (35, 48, 49), and with a never reported longer length of hospital stay.

As the ratio between the amount of circulating immune cell type may refine the definition of malnutrition, we also analyzed the predictive role of NLR, LMR and PLR. We showed a clear association between NLR and LMR with a longer length of hospital stay, in agreement with previous data (36, 37, 50). Furthermore, OS seemed to be correlated only with NLR, in contrast to Zhang et al., where patients with shorter OS had both elevated NLR and PLR.

Furthermore, few studies have focused on the association between the modulation of postoperative hematological parameters and the prediction of malnutrition. We reported a significant association between patients at high risk of malnutrition (defined by a MUST score ≥1 or albumin levels <35 mg/dL) before surgery and a greater decrease in hemoglobin (i.e., anemia) in early PODs. Anemia, which is common after surgery, is associated with increased risk of complications and decreased overall survival (31). In addition, blood transfusions are associated with increased mortality, morbidity, prolonged hospital stay, risk of anastomotic leakage, and worse oncologic outcomes (51, 52). Indeed, we found a direct association between malnutrition and anemia, in patients characterized by higher complication rates, greater need for nutritional support (both parenteral and enteral) and subsequent longer hospital stay. All these outcomes result in higher hospital costs and lower survival rates and quality of life (53, 54).

## 5 Conclusion

This retrospective cohort study analyzed the correlation of various nutritional and inflammatory parameters with postoperative outcomes in patients with gastrointestinal cancer. Our results highlighted the prognostic role of MUST score, albumin and PNI. Inflammatory parameters seemed to be less predictive for most of the outcomes, but NLR was statistically associated with 1-year OS. Furthermore, assessing the independence of biomarkers as prognostic factors by multivariate analysis, we confirm MUST, NLR and PLR as predictors of differential OS, whereas postoperative complications seem to be influenced only by nutritional factors. In an evolving scenario, if these cut-offs are confirmed by other studies, these predictive parameters should be included in the clinical routine to address to more tailored nutritional interventions. Further research on the efficacy of different nutritional approaches in improving these parameters is highly desirable to improve patient outcomes.

## Data Availability

The raw data supporting the conclusions of this article will be made available by the authors, without undue reservation.

## Supplementary material

SUPPLEMENTARY FIGURE 1 Timeline of the study. The timeline reports data collected at the 4 pivotal time points (in black) and at the three routinely-performed postoperative hematic withdraws (in red).

## Glossary

MUST: Malnutrition Universal Screening Tool
PNI: Prognostic Nutritional Index
NLR: Neutrophil-to-Lymphocyte Ratio
PLR: Platelet-to-Lymphocyte Ratio
LMR: Lymphocyte-to-Monocyte Ratio
GC: Gastrointestinal Cancer
NRS-2002: Nutritional Risk Screening 2002
BMI: Body Mass Index
OS: Overall Survival
PODs: postoperative days
GLM: General Linear Models
OR(s): Odd Ratio(s)
CRC: Colorectal cancer
M+: gastrointestinal Metastatic cancer
UGI: Upper Gastroesophageal cancers
HPB: Hepatobiliary cancer
PAN: Pancreatic cancer
PN: parenteral nutrition
EN: enteral nutrition
NA: not applicable
WBC: White Blood Cells
CT: Chemotherapy
RT: Radiotherapy
