# Supplementary figures and images for: Clinical significance of preoperative nutrition and inflammation assessment tools in gastrointestinal cancer patients undergoing surgery: a retrospective cohort study
Source: Front Nutr. 2025 May 19;12:1551048. doi: 10.3389/fnut.2025.1551048 (PMC12127174; doi:10.3389/fnut.2025.1551048)

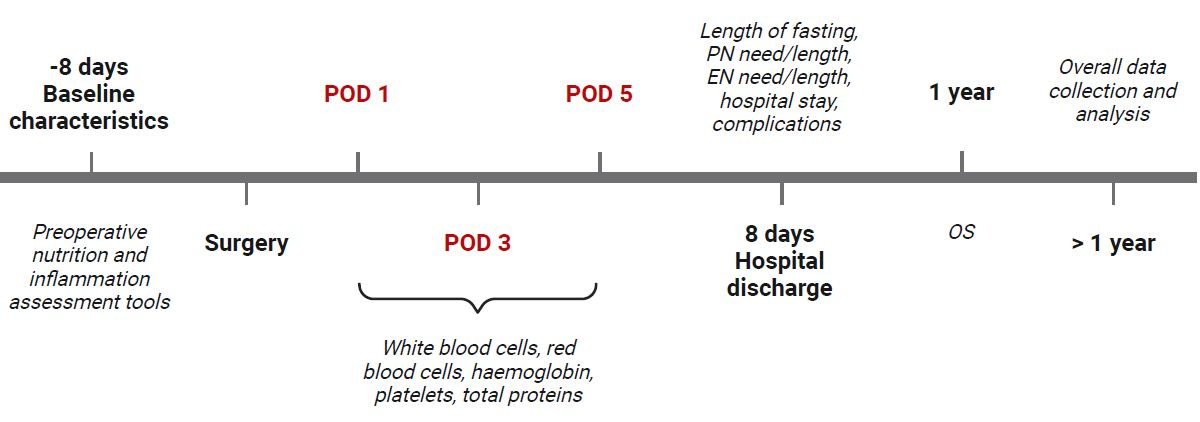

Supplement: SUPPLEMENTARY FIGURE 1 — Timeline of the study. The timeline reports data collected at the 4 pivotal time points (in black) and at the three routinely-performed postoperative hematic withdraws (in red). [file Figure_1.TIFF]
